# Influence of Target Surface BRDF on Non-Line-of-Sight Imaging

**DOI:** 10.3390/jimaging10110273

**Published:** 2024-10-29

**Authors:** Yufeng Yang, Kailei Yang, Ao Zhang

**Affiliations:** 1 College of Automation & Information Engineering, Xi’an University of Technology, Xi’an 710048, China; yangyufeng@xaut.edu.cn (Y.Y.); 2210321184@stu.xaut.edu.cn (A.Z.); 2 Xi’an Key Laboratory of Wireless Optical Communication and Network Research, Xi’an 710048, China; 3 Nanjing Institute of Multi-Platform Observation Technology, Nanjing 211500, China

**Keywords:** bidirectional reflection distribution function, deep learning, spatial target material, non-line-of-sight imaging

## Abstract

The surface material of an object is a key factor that affects non-line-of-sight (NLOS) imaging. In this paper, we introduce the bidirectional reflectance distribution function (BRDF) into NLOS imaging to study how the target surface material influences the quality of NLOS images. First, the BRDF of two surface materials (aluminized insulation material and white paint board) was modeled using deep neural networks and compared with a five-parameter empirical model to validate the method’s accuracy. The method was then applied to fit BRDF data for different common materials. Finally, NLOS target simulations with varying surface materials were reconstructed using the confocal diffusion tomography algorithm. The reconstructed NLOS images were classified via a convolutional neural network to assess how different surface materials impacted imaging quality. The results show that image clarity improves when decreasing the specular reflection and increasing the diffuse reflection, with the best results obtained for surfaces exhibiting a high diffuse reflection and no specular reflection.

## 1. Introduction

Non-line-of-sight (NLOS) imaging is a technique used to capture scenes or objects that are out of sight and not directly observable. Depending on whether a controlled light source is used, NLOS imaging techniques can be classified into two types: One is active NLOS imaging, which captures the shape of a hidden object by actively emitting a light ray that eventually captures the shape of the hidden object through scattering and diffuse reflection information after interacting with the occluder and the hidden object. The other is passive NLOS imaging, which does not rely on a controlled light source but, rather, utilizes ambient light or the hidden object’s luminescence to capture the shape of a hidden object by capturing the hidden object on the occluder object via capturing the projection information formed by the object on the occluding object to reconstruct the object [1]. In the field of NLOS imaging research, A. Velten et al. [2] utilized time-of-flight imaging to detect the 3D shape of an object beyond the line of sight. However, this method faces the problems of large, complex data processing, expensive equipment, and time consumption. T. Maeda et al. [3] realized passive NLOS imaging using infrared light, but the quality of the imaging was not satisfactory due to the difficulty of light focusing. The introduction of the Light-Cone Transform algorithm [4], which allows the simplification of the optical path transmission model through the confocal mode and assumes the target to be a Lambertian body, improves the imaging efficiency and quality. D.B. Lindell et al. [5] proposed a confocal diffuse tomography (CDT) imaging technique to overcome the limitations of scattering on the optical system, which can recover the 3D shape of the hidden object and achieve imaging in complex environments. However, achieving high resolution is difficult, and the reflection characteristics of the target material are single. Ye et al. [6] reduced the number of scanning points by using the compressed perception method, which, in turn, saves the time required for the data acquisition and accelerates the speed of imaging. In China, some attempts have been made to improve the algorithm of NLOS imaging technology. Pei et al. [7] utilized SPAD arrays to provide the NLOS imaging system the ability of “single transmitter and multiple receivers”, which improves the imaging speed. Sun et al. [8] utilized a novel learning-based solution to improve the NLOS imaging problem, which can be applied to scenarios with low signal-to-noise ratios. The methodology comprises two key designs: learnable path compensation (LPC) and the adaptive phasor field (APF). However, NLOS simulation studies usually set the target as a Lambertian body, which leads to a single reflection characteristic and hinders the true reflection of a complex environment. This simplification limits the practical application and development of NLOS. Hence, the bidirectional reflection distribution function (BRDF) is introduced into NLOS imaging in this paper.

BRDF, which is mainly determined by the roughness, dielectric constant, surface texture, polarization, and incident wavelength of the target surface, characterizes the distribution of the reflected energy of incident light in hemispherical space [9]. In practice, the measurement of BRDF is limited by its inability to obtain accurate values under arbitrary incidence and observation conditions [10,11,12]. The BRDF models are categorized into empirical, physical, and data-driven models. Empirical models can effectively capture the reflective behavior of common materials, such as the Lambertian model, Phong model [13], five-parameter model [14,15], and Minnaert model [16], but their applications are limited, and describing the reflective properties of complex materials is difficult. Physical models are based on the laws of physics, such as the Torrance–Sparrow model [17] and the Maxwell–Baird model [18]. Fu et al. [19]. proposed an improved pBRDF model, which improved the relative error in the polarization characteristics of the target surface by means of parameter inversion. Ma et al. [20]. measured the spectral polarization BRDF of copper materials in the visible light band with a self-designed device, and they established an exponential model to describe these properties. However, the lack of measurement of bidirectional reflection distribution functions for other different materials limits the generalization ability of the model. Although these models are highly accurate, they involve high computational complexity and cumbersome parameter settings. By contrast, data-driven models learn the reflective properties of materials from a large amount of measurement data via methods such as machine learning [21]. The data-driven model does not require an in-depth understanding of the microstructure and physical properties of the material, which reduces the threshold of model construction.

In summary, this paper adopts a deep learning method to model the target BRDF. Then, the BRDF is introduced into the NLOS imaging scene, and the CDT imaging algorithm in NLOS imaging is simulated based on the principles of the diffusion equation, confocal setup, and imaging model. Next, 3D reconstruction is performed through seven typical BRDF types of NLOS targets and three representative examples of NLOS targets under the deep learning approach. Finally, the effects of different target surface materials on the NLOS imaging quality are analyzed by constructing a convolutional neural network to classify the reconstruction results of different BRDF targets.

## 2. Materials and Methods

### 2.1. BRDF Definition and Principle

BRDF is defined as the relative magnitude of the radiant luminance ${dL}_{s}(\theta_{i},\varphi_{i},\theta_{s},\varphi_{s})$ along the direction of λ,$\theta_{i}$, and $\varphi_{i}$ to the radiant illuminance, ${dE}_{i}(\theta_{i},\varphi_{i})$, incident on the surface of the object along the direction of ($\theta_{i}$, $\varphi_{i}$), with the following expression:

(1) $$f_{r}\left(\lambda ,\theta_{i},\varphi_{i},\theta_{s},\varphi_{s}\right)=\frac{dL_{s}\left(\lambda ,\theta_{i},\varphi_{i},\theta_{s},\varphi_{s},E_{i}\right)}{dE_{i}\left(\lambda ,\theta_{i},\varphi_{i}\right)}$$

where *λ* is the wavelength, $\theta_{i}$ is the incident zenith angle, $\theta_{s}$ is the scattered zenith angle, $\varphi_{i}$ is the incident azimuth angle, $\varphi_{s}$ is the scattered azimuth angle, and $f_{r}$ is the BRDF of the surface element.

**Figure 1:**
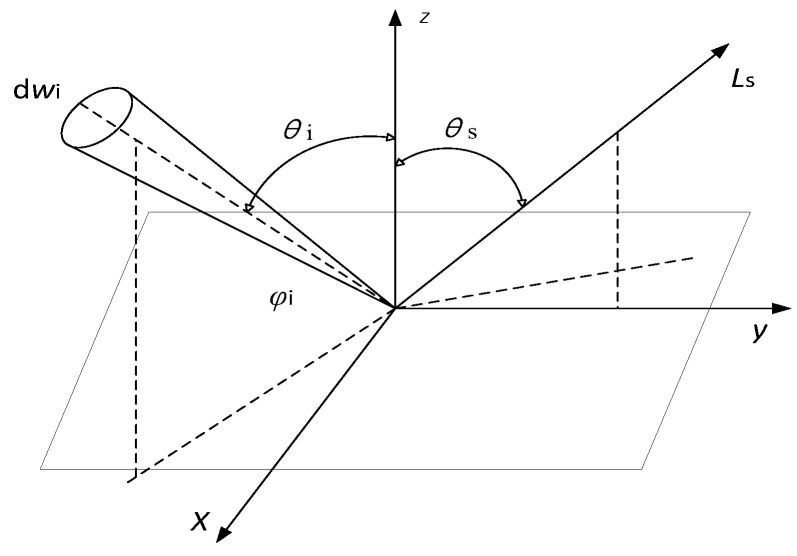
Geometric diagram of BRDF.

BRDF depends on factors such as the dielectric constant, radiation wavelength, and surface roughness, and its geometry is shown in Figure 1:

According to the defining equation of BRDF, the BRDF data to be measured include the incident zenith angle, $\theta_{i}$, the incident azimuth angle, $\varphi_{i}$, the scattered zenith angle, $\theta_{s}$, the scattered azimuth angle, $\varphi_{s}$, and the value of BRDF.

### 2.2. Diffusion Equation

According to the Boltzmann radiation transport equation, the flux rate, φ(r,t), of the diffused photons satisfies the diffusion equation when the collimated pulsed beam is incident on the surface of a semi-infinite homogeneous tissue plate or a finite homogeneous tissue plate with the following expression:

(2) $$\frac{1}{c}\frac{\partial }{\partial t}\varphi \left(r,t\right)-D\nabla^{2}\varphi \left(r,t\right)+\mu_{a}\varphi \left(r,t\right)=S\left(r,t\right)$$

(3) $$D={\left\{3\left[\mu_{a}+\left(1-g\right)\mu_{s}\right]\right\}}^{-1}$$

where c is the velocity of light in the tissue, t is the time, D is the diffusion coefficient, and S(r,t) is the photon source; $\mu_{a}$ and $\mu_{s}$ are the linear absorption and scattering coefficients, respectively, and *g* is the average cosine of the scattering angle.

(4) $$\varphi \left(r,t\right)=c{\left(4\pi Dct\right)}^{-3/2}\exp\left(-\frac{r^{2}}{4Dct}-\mu_{a}ct\right)$$

(5) $$Z_{0}={\left[\left(1-g\right)\cdot \mu_{s}\right]}^{-1}$$

A common approximation uses an extrapolated boundary condition for which the diffusive intensity is assumed to be zero on a flat surface in an extrapolation distance, $z_{e}$, away from either side of the slab. For a slab of thickness $z_{d}$, this condition states that the diffusive intensity is zero at $z=-z_{e}$ and ${z=z}_{d}+z_{e}$, where the value of $z_{e}$ depends on the amount of internal reflection for the diffusive intensity due to the refractive index mismatch at the medium–air interface.

**Figure 2:**
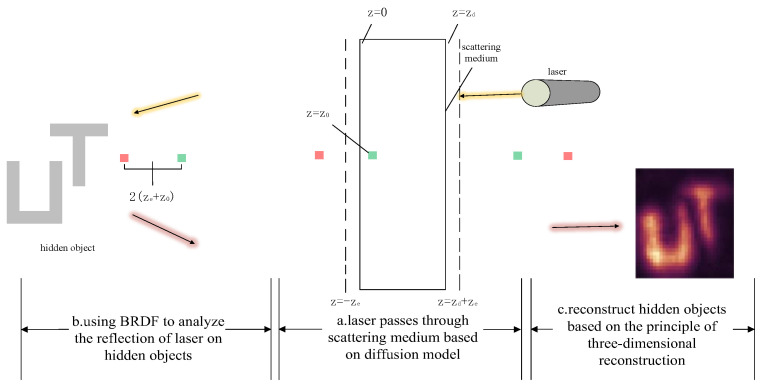
Model of CDT imaging.

Hence, the extrapolated boundary condition can be satisfied by placing a positive and negative dipole source about $z=-z_{e}$ such that the total diffusion intensity at the extrapolated distance is zero. However, a single dipole source does not satisfy the boundary condition at ${z=z}_{d}+z_{e}$. Instead, an infinite number of dipole sources are needed, for among which the dipole at the near interface ($z=-z_{e}$) is mirrored by the dipole at the far interface and then again by the dipole at the far interface, and so on, as illustrated with the small squares in Figure 2.

The locations of the positive and negative sources are as follows:

(6) $$\begin{array}{l} z_{+,i}=2i\left(z_{d}+2z_{e}\right)+z_{0}\\ z_{-,i}=2i\left(z_{d}+2z_{e}\right)-2z_{e}-z_{0}\\ i=0,\pm 1,\pm 2,\cdots \end{array}$$

Finally, the resulting solution to the diffusion equation is as follows:

(7) $$\begin{array}{l} \varphi \left(t,r_{0},r_{1}\right)=\frac{1}{2{\left(4\pi Dc\right)}^{3/2}t^{5/2}}\exp\left(-\mu_{a}ct-\frac{{\left(r_{1,x}-r_{0,x}\right)}^{2}+{\left(r_{1,y}-r_{0,y}\right)}^{2}}{4Dct}\right)\cdot \\ \sum_{i=-\infty }^{\infty }\left[\left(z_{d}-z_{+,i}\right)\exp\left(-\frac{{\left(z_{d}-z_{+,i}\right)}^{2}}{4Dct}\right)-\left(z_{d}-z_{-,i}\right)\exp\left(-\frac{{\left(z_{d}-z_{-,i}\right)}^{2}}{4Dct}\right)\right] \end{array}$$

where *Φ* is the power transmitted through the slab per unit area, $r_{0}\in \Omega_{0}=\left[\left.r_{0,x},r_{0,y},\left.r_{0,z}\right)\in R\times R\times R|r_{0,z}=0\right]\right.$ is the position illuminated via the laser and imaged via the detector, and $r_{1}\in \Omega_{z_{d}}=\left[\left.r_{1,x},r_{1,y},\left.r_{1,z}\right)\in R\times R\times R|r_{1,z}=z_{d}\right]\right.$ is a spatial position on the far side of the scattering medium.

### 2.3. Imaging Model

The complete CDT imaging model consists of three parts: laser diffusion through the scatterer, free-space propagation hitting the hidden object, and reflection via the hidden object and passing through the scatterer again.

The measurement model is as follows:

(8) $$\begin{array}{l} \tau (t,r_{0})=\int_{\Omega_{z_{d}}}\int_{0}^{\infty }\left[\underset{r_{0}\to r_{1}}{\underset{︸}{\int_{\Omega_{z_{d}}}\int_{0}^{\infty }\phi \left(\left.t\prime \prime -t\prime ,r_{0},r_{1}\right)\right.}}\right.\\ \left.\begin{array}{c} \;\;\;\;\;\underset{I(t\prime ,r_{1},r_{2}):r_{1}\to x\to r_{2}}{\underset{︸}{\left[\left.\int_{\psi }f(x,r_{1})f(x,r_{2})\sigma (ct\prime -\left\|x-r_{1}\right\|-\left\|x-r_{2}\right\|)dx\right]\right.}}dt\prime dr_{1} \end{array}\right]\\ \begin{array}{c} \;\;\;\;\;\underset{r_{2}\to r_{0}}{\underset{︸}{\phi \left(\left.t\prime \prime -t\prime ,r_{0},r_{2}\right)\right.dt\prime \prime dr_{2}}} \end{array} \end{array}$$

The measured value $\tau \left(t,r_{0}\right)$ is an integral of three parts: the first part is the diffusion of light from $r_{0}$ to $r_{1}$ in the scattering medium, the second part is the free space propagation of the light from $r_{1}$ to the point x on the hidden object and back to another $r_{2}$, and the third part is the diffusion of the light from $r_{2}$ to $r_{0}$ through the scattering medium.

In this paper, the confocal setting as a scheme, namely, $r_{1}\approx r_{2}$, is used to simplify the imaging model. The expression is as follows:

(9) $$\hat{\tau }\left(t,r_{0}\right)=\varphi \left(t,r_{0},r_{1}\right)*\varphi \left(t,r_{0},r_{1}\right)*I\left(t,r_{1},r_{1}\right)=\bar{\varphi }*I$$

(10) $$I\left(t,r_{1},r_{1}\right)=\int_{\psi }f\left(x,r_{1}\right)f\left(x,r_{1}\right)\delta \left(ct-2\left\|x-r_{1}\right\|\right)dx$$

where the function *f* is the albedo from a point on the hidden object to a point on the boundary of the scattering medium, and the δ function of the constraint time is $\delta (ct-2|\left|x-r_{1}\right||)$.

For the inverse process of the above model, because the real imaging model contains noise terms, it needs to be deconvolved through Wiener filtering, which leads to a closed-form solution with the following expression:

(11) $$\hat{I}=g\left(t\right)*\hat{\tau }\left(t,r_{0}\right)$$

(12) $$G\left(f\right)=\frac{{\hat{\bar{\Phi }}}^{*}}{{\left|\hat{\bar{\Phi }}\right|}^{2}+\frac{1}{R_{\mathrm{SN}}}}$$

where the superscript * represents the complex conjugate, and RSN is the signal signal-to-noise ratio. Combining Equations (11) and (12) derives the frequency domain representation of $\hat{I}$, and then its inverse Fourier transform estimates the value of *I*. The expression is as follows:

(13) $$\hat{I}=F^{-1}\left\{\frac{{\hat{\bar{\Phi }}}^{*}}{{\left|\hat{\bar{\Phi }}\right|}^{2}+\frac{1}{R_{\mathrm{SN}}}}\cdot F\left[\hat{\tau }\left(t,r_{0}\right)\right]\right\}$$

(14) $$\rho =A^{-1}F^{-1}\left[\frac{{\hat{\bar{\Phi }}}^{*}}{{\left|\hat{\bar{\Phi }}\right|}^{2}+\frac{1}{R_{\mathrm{SN}}}}\right]F\left[\hat{\tau }\left(t,r_{0}\right)\right]$$

Thus, the albedo of the hidden object is obtained, and finally, the geometry of the hidden object is reconstructed.

## 3. Results

### 3.1. Deep Neural Network Target BRDF Model Construction

Deep learning is a machine learning method based on artificial neural networks for data analysis and pattern recognition. Artificial neural networks mimic the structure and function of neurons in the human brain and contain an input layer, a hidden layer, and an output layer. The input layer receives the data, the hidden layer processes the data, and the output layer generates the final result.

(1) Input and output of the model

In this paper, the input layers are incident zenith angle $\theta_{i}$, incident azimuth angle φi, scattering zenith angle $\theta_{s}$, and scattering azimuth angle $\varphi_{s}$, and the output layer is the simulation value f of BRDF.

(2) Model network structure selection

Due to the numerous factors that determine the distribution of BRDF, the premise of modeling BRDF is to extract relevant features adequately from experimental data. Deep neural networks connect nodes through layers, the output of the previous hidden layer becomes the input of the next hidden layer, and the cycle repeats. In this process, the effectiveness of feature extraction increases as the number of hidden layers increases. However, extremely many layers weaken the learning ability of the hidden layer and affect the accurate extraction of features. Hence, in this paper, a deep neural network containing five hidden layers is constructed, and the numbers of neurons in these hidden layers are 20, 30, 20, 10, and 10, in that order.

(3) Activation function

In this paper, the fully connected neural network is used as a deep neural network, and each layer of the fully connected neural network can be calculated by multiplying the weight matrix, *W*, with the neuron vector, *x*, and then adding it to the bias term, *b*, as shown below:

(15) y=W⋅x+b

Because the modeling outputs result in BRDF simulation values, the value of the activation function must be greater than 0. If its value is less than 0, the network is in a state of inhibitory activity, so the activation function is selected as the ReLU activation function.

(4) Loss function and optimizer selection

The loss function is the only measure of the accuracy of the model. If the value of the loss function is smaller, then the degree of deviation is smaller. The loss function expression is as follows:

(16) $$E=\frac{{\left|y_{prer}-f_{0}\right|}^{2}}{{\left|f_{0}\right|}^{2}}$$

where *E* is the difference value between the simulation result and the measurement result, $y_{prer}$ is the simulation result, and $f_{0}$ is the actual measurement result.

To avoid falling into a local optimum when debugging parameters, unsupervised learning is used as the training method for a deep neural network, and the DropOut optimization algorithm is introduced.

(5) Regularization method

Regularization techniques can reduce the complexity of deep neural network models and prevent model overfitting. In this paper, the L1 regularization method with the following expression is used:

(17) $$L=\frac{{\left|y_{prer}-f_{0}\right|}^{2}}{{\left|f_{0}\right|}^{2}}+\lambda \sum_{i=1}^{n}\left|\theta_{i}\right|$$

where *L* denotes regularization, and $\lambda \sum_{i=1}^{n}\left|\theta_{i}\right|$ is the regular term.

**Figure 3:**
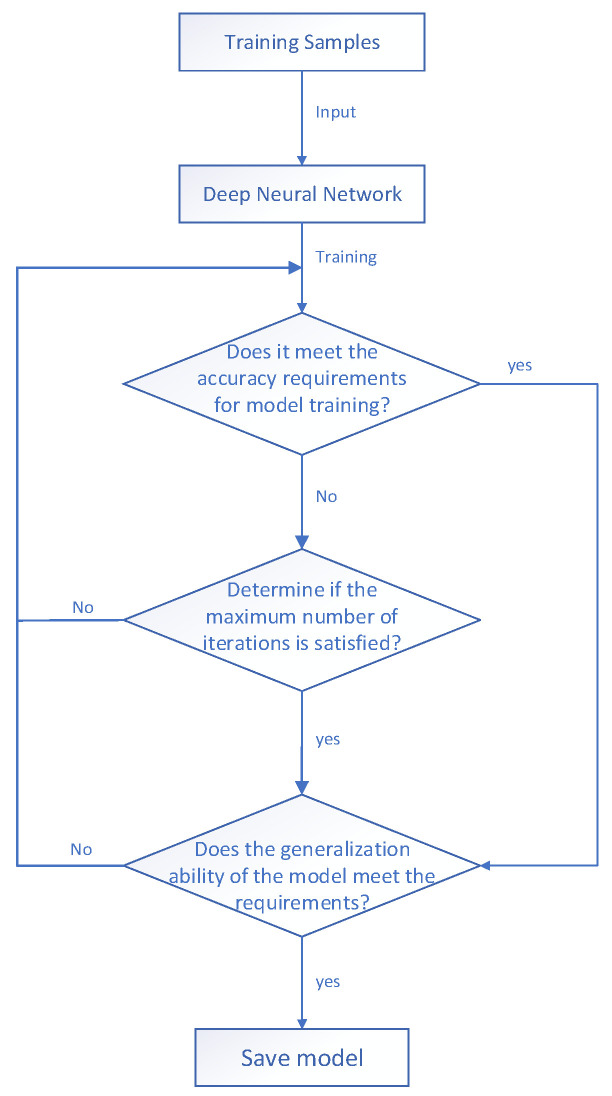
Modeling of BRDF based on deep neural network.

Based on the above, the target BRDF modeling derived from a deep neural network can be demonstrated in a flowchart, as shown in Figure 3.

The surface BRDF data of different common objects are measured at incident zenith angles of 30°, 45°, and 60°, and the amount of data collected for each object is 1000. For the surface BRDF experimental data of a certain object, the scheme adopted is to select it randomly first, take part of the experimental data as a training sample and the rest as a test sample, and then take the training samples as inputs to construct the BRDF model.

### 3.2. Target Imaging Classification Based on Deep Learning

The reconstructed images of hidden objects with different target object materials are different. The data set for target classification can be obtained after numerous simulations. Combined with the features of the reconstruction results, the images can be classified into three, namely, clear, clearer, and fuzzy, and they are labeled in order as 1, 2, and 3, respectively. When classifying the target through the use of deep learning convolutional neural networks, the width of the input layer is generally H, the height is generally W, and the number of channels is D. In the case of RGB images, the value of D is taken as 3. The convolutional layer performs convolutional operations on different images and then extracts the image features and finally merges all the different image features collected.

**Figure 4:**
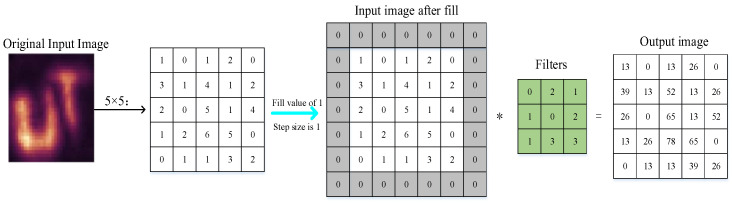
Schematic diagram of convolution operation.

With the 5 × 5 raw input image and the 3 × 3 filter taken as an example, and the fill value and step size are set to 1, the convolution operation is shown in Figure 4.

The activation function used in this paper at the fully connected layer is the same as the activation function used to build the target BRDF model with deep learning, which is also the ReLU function. In this manner, not only can the nonlinear component of the convolutional neural network be increased but also the gradient can disappear when the input data is large or small; that is, the problem of a gradient value close to 0 can be avoided.

The output layer treats the normalization function as the result of the classification output with the following expression:

(18) $$p_{i}=\frac{e^{a_{i}}}{\sum_{k=1}^{N}e^{a_{k}}}$$

The method of calculating probability is to map the output values of many inputs to the interval of 0–1, and the classification corresponding to the largest probability value is the final result.

The specific expression of the loss function used in this paper is cross-entropy because the loss function in the form of cross-entropy can measure the difference between the simulation result and the actual label, which often appears in the problem of target classification. The specific formula for the cross-entropy loss function is as follows:

(19) $$L\left(y,p\right)=-\sum_{i}y_{i}\log\left(p_{i}\right)$$

where *L* is the loss function, and $y_{i}$ is the true value corresponding to a particular image. $p_{i}$ takes values in the range of 0–1, and the sign in front of the cumulative sign indicates that, when the probability of the simulation result, $p_{i}$, is larger, the value of the loss function is lower.

## 4. Analysis of Results

### 4.1. BRDF Model Validation Analysis

To verify the validity and accuracy of the BRDF model, the five-parameter BRDF model simulation modeling was used, and then its modeling results were analyzed and compared with those of the deep neural network BRDF model.

**Figure 5:**
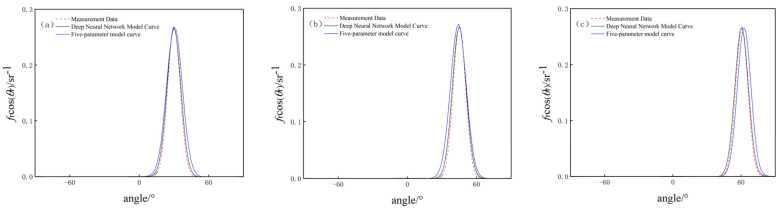
Comparison of BRDF data for aluminized insulation material under two models. (**a**) $\theta_{i}$ = 30°; (**b**) $\theta_{i}$ = 45°; (**c**) $\theta_{i}$ = 60°.

**Figure 6:**
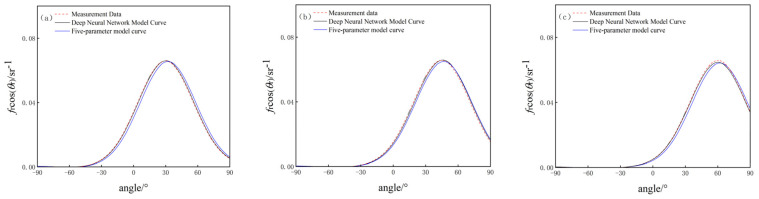
Comparison of BRDF data for white paint board under two models. (**a**) $\theta_{i}$ = 30°; (**b**) $\theta_{i}$ = 45°; (**c**) $\theta_{i}$ = 60°.

Aluminized insulation material performs stronger specular reflection, while white paint board involves stronger diffuse reflection, according to the experimental data of their surface BRDF, in which the incident zenith angles are taken as 30°, 45°, and 60°, and the relative azimuthal angles are all taken as 0°. The results of the surface BRDF of the two materials are obtained through the deep neural network modeling and the five-parameter empirical modeling methods, as shown in Figure 5 and Figure 6.

(1) Comparison of BRDF data of aluminized insulation material

The training error is 0.0457, the test error is 0.0339, the average agreement between the deep neural network model and the measured data is 95%, and the average agreement between the five-parameter model and the measured data is 84%.

According to the comparison results of the BRDF measurement data with the model data under the deep neural network model and the five-parameter model for the aluminized insulation material, when the incident zenith angle $\theta_{i}$ is different, the BRDF value of the target object surface simulated via the deep neural network model is more compatible with the measured value, and the fitting effect of this model is better than that of the five-parameter model, which indicates that, for the object with strong specular reflection, the target BRDF model based on deep learning is more compatible with the measurement data. The learning target BRDF model has a better simulation ability for objects with strong specular reflection.

(2) Comparison of BRDF data of white paint board

The training error is 0.0372, the test error is 0.0429, the average agreement between the deep neural network model and the measurement data is 92%, and the average agreement between the five-parameter model and the measurement data is 83%.

According to the comparison results of the BRDF measurement data with the model data under the deep neural network model and the five-parameter model for the white paint board, for the objects with strong diffuse reflections, the fitting effect of the deep neural network model is also better than that of the five-parameter model, which indicates that, for the objects with strong diffuse reflections, the simulation capability of the target BRDF model based on deep learning is still better than that of the five-parameter model.

**Figure 7:**
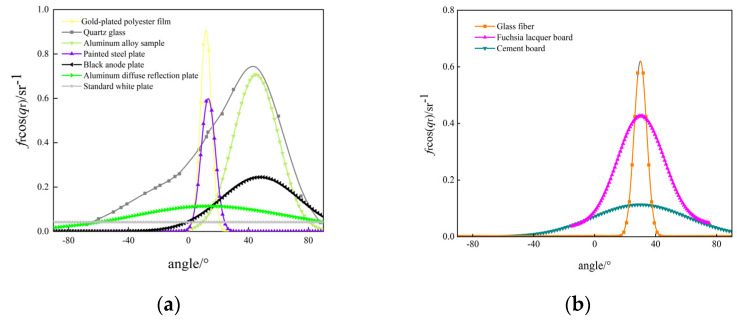
BRDF fitting results for different target objects. (**a**) BRDF fitting results for seven target objects; (**b**) BRDF for three instances.

Then, by using the deep neural network BRDF model, the BRDFs of seven target objects are simulated and fitted, and the results are shown in Figure 7. In Figure 7a, the fitted curves of the seven different target objects represent four types of typical BRDFs: The fitted curves of the gold-plated polyester film and the painted steel plate have narrow shapes but high peaks, representing the type with a strong specular reflection part. The fitted curves of the quartz glass and the aluminum alloy samples have wide shapes and high peaks, representing the type with a strong specular and diffuse reflection part. The fitted curves of the black anode plate and aluminum diffuse reflection plate have wide shapes but low peaks, which represent the class with strong diffuse reflection part. The fitted curves of the standard white plate are straight lines, which represent the class with no specular reflection part, when the target object is a Lambertian body.

To verify the above analysis, the BRDF data plots of glass fiber with a strong specular reflection part, fuchsia lacquer board with a strong specular reflection part and a strong diffuse reflection part, and cement board with a strong diffuse reflection part, which are obtained from the BRDF measurement data of the three examples, are given, as in Figure 7b, where the specular reflection part of the glass fiber is stronger than that of the two other materials, and the peak value of their BRDF curves is the highest. A large part of the reason for this is that the refractive index of glass fiber is smaller, and the smaller the refractive index is, the smaller the absolute value of the dielectric constant is, resulting in a larger echo energy on the surface of the object, which in turn leads to a higher peak value.

### 4.2. Target CDT Imaging Reconstruction

The hidden scene consists of the letters “UT” located about 50 cm behind the scatterer, the thickness of the scattering medium is 2.54 cm, the refractive index is 1.12, and other important simulation parameters are set as shown in Table 1.

**Figure 8:**
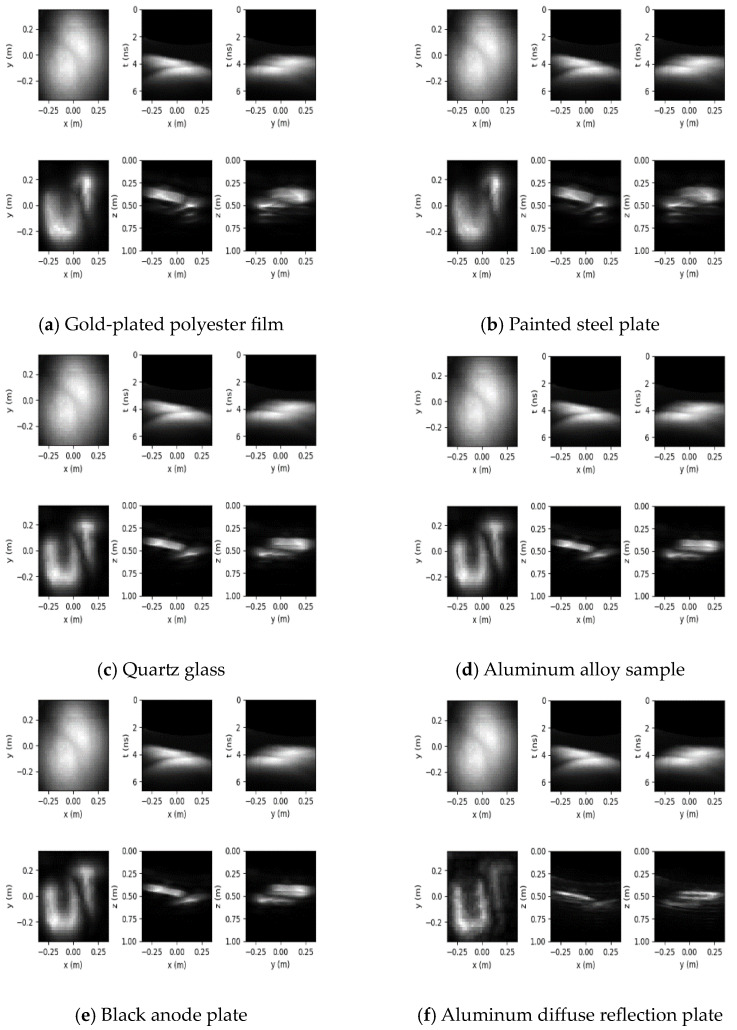

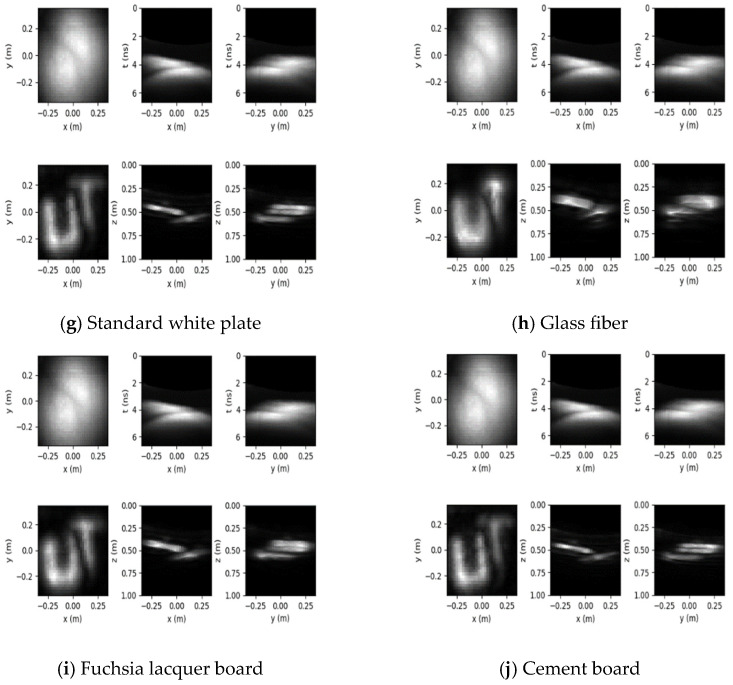
Reconstruction results of different objects.

Based on the principle of 3D reconstruction, the Python 3.6 software was used to reconstruct the hidden objects of seven kinds of objects representing four types of typical BRDFs and three instance objects, and the reconstruction results are shown in Figure 8.

The 3D reconstruction results for the different hidden objects are presented in Figure 8, where the first row of each subplot shows the measurements and their x–t and y–t slices, and the second row shows the reconstruction results and their x–z and y–z slices.

### 4.3. Imaging Classification of Different BRDF Surface Targets

The data set constructed in this paper contains 1000 images, of which 200 and 800 were used for validation and training, respectively. Fast Fourier Transform is a frequently used tool in problems related to frequency-domain analysis to transform an image from the spatial to the frequency domain. In the frequency domain, the high frequencies correspond to the detailed parts of the image, and the low frequencies correspond to the general shape of the image.

Because the detail part implies a large amount of edge information, its higher variance indicates a higher degree of deviation, which in turn means a higher intensity of information and a clearer image. Hence, in this paper, Fast Fourier Transform is performed on 1000 images in the data set, and the variance of the edge information in the high frequency range is calculated to determine the clarity of the image. After computational evaluation, in these 1000 images, 193, 514, and 293 images are labeled 1, 2, and 3, respectively.

**Figure 9:**
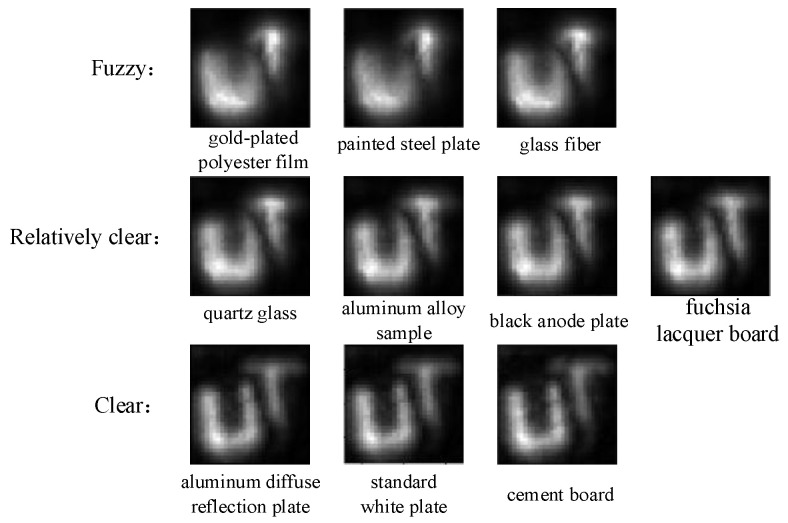
Classification results of different BRDF surface targets.

In this paper, PyTorch is selected as the framework for deep learning, the number of training times is set to 10, and the cross-entropy loss function is taken as the optimization object. After the training is completed, the reconstructed images Figure 8a–j of the above 10 objects are input into the classification network, one object corresponds to one image, 10 images are not labeled, and finally, the classification results of different BRDF surface targets are obtained, as shown in Figure 9.

Figure 9 shows 10 types of objects divided into three using convolutional neural networks: Gold-plated polyester film, painted steel plate, and glass fiber belong to the category labeled as 3, which is fuzzy. Quartz glass, aluminum alloy sample, black anode plate, and fuchsia lacquer board belong to the category labeled as 2, which is relatively clear. aluminum diffuse reflection board, standard white plate, and cement board belong to the category with a label of 1, which is clear.

## 5. Conclusions

In this paper, a target BRDF model suitable for the scattering characteristics of object materials based on deep learning was constructed, and this method was used to model the BRDF of two surface materials (aluminized insulation material and white paint board). The results indicate that deep learning models can effectively simulate the BRDF of the target object surface, whether it is an object with strong specular reflection or an object with strong diffuse reflection. The deep learning model outperforms the five-parameter model in fitting performance, more accurately describes the target scattering characteristics, and verifies the accuracy and effectiveness of this method. Then, this method was used to fit the surface BRDF data of seven materials (gold-plated polyester film, quartz glass, an aluminum alloy sample, painted steel plate, black anode plate, aluminum diffuse reflection plate, and standard white plate). The BRDF data were analyzed and compared with three examples (glass fiber, fuchsia lacquer board, and cement board). Finally, the confocal diffusion tomography algorithm was used to simulate and reconstruct NLOS targets of these 10 different surface materials. CDT imaging reconstruction and classification analysis revealed that, as the proportion of specular reflection in the object material decreased, the effect of NLOS reconstruction remarkably improved. The BRDF distribution map shows that, when the incident angle is the same, the stronger the specular reflection of the object, the larger the highlight area generated on the surface and the greater the effect on intrinsic texture and other information, leading to a decrease in reconstruction quality. Therefore, for target objects with different surface materials, the smaller the proportion of their specular reflection, the better the reconstruction effect.

## Figures and Tables

**Table 1:** Other important simulation parameters of non-line-of-sight imaging systems.

Name	Value
Scanning area/$m^{2}$	0.09
Absorption coefficient of the scatterer medium/${cm}^{-1}$	$5.30\times 10^{-3}$
Scattering coefficient of the scatterer medium/${cm}^{-1}$	2.62
Distance between laser and scanning center/m	1.3
Pulse repetition rate/MHz	10
Pulse width of the laser/ps	35
Wavelength of the pulsed laser/nm	532
Average power of the pulsed laser/mW	400

## Data Availability

The original contributions presented in this study are included in the article, further inquiries can be directed to the corresponding author.
